# From prevalence to prognosis: the clinical relevance of depression and anxiety in COPD

**DOI:** 10.1080/02813432.2026.2738628

**Published:** 2026-10-13

**Authors:** Josep Montserrat-Capdevila

**Affiliations:** ^a^ Family and Community Medicine Teaching Unit, Catalan Health Institute (ICS), Lleida, Spain; ^b^ Faculty of Medicine, University of Lleida, Lleida, Spain; ^c^ Fundació Institut Universitari per a la Recerca a l’Atenció Primària de Salut Jordi Gol i Gurina (IDIAPJGol), Barcelona, Spain

**Dear Editor**

We read with great interest the recent article by Öfverholm et al. [1] reporting a high prevalence of depression and anxiety among younger patients with chronic obstructive pulmonary disease (COPD) in Swedish primary care. The authors should be congratulated for highlighting an important but often under-recognised aspect of COPD management in primary care.

Their findings demonstrate that depression and anxiety are frequent comorbidities, particularly among younger patients, women, current smokers, and individuals with greater symptom burden. These observations reinforce the need for a comprehensive, patient-centred approach to COPD management.

We would like to further expand the clinical implications of these findings. Beyond their high prevalence, depression and anxiety also have important prognostic implications. In a prospective population-based cohort of patients with COPD managed in primary care, we previously demonstrated that symptoms of depression and anxiety, assessed using the validated Hospital Anxiety and Depression Scale (HADS), were independently associated with a significantly increased risk of frequent COPD exacerbations over two years of follow-up, even after adjustment for relevant clinical confounders [2].

The cross-sectional findings reported by Öfverholm et al. [1] complement, rather than overlap with, our prospective observations. Together, these studies strengthen the evidence that systematic assessment of psychological comorbidity should become an integral component of routine COPD care in primary care.

An additional methodological consideration is that our study used the validated HADS questionnaire, whereas Öfverholm et al. [1] relied on self-reported depression and anxiety. Although both approaches provide valuable information, validated screening instruments may improve comparability across studies and facilitate risk stratification in clinical practice.

Future prospective studies should evaluate whether systematic screening and timely management of depression and anxiety can reduce COPD exacerbations, healthcare utilisation, and improve patients’ quality of life.

We congratulate Öfverholm et al. [1] on their valuable contribution and believe that integrating evidence on both prevalence and prognosis further supports a more holistic approach to COPD management in primary care.
